# Housing instability and cardiometabolic health in the United States: a narrative review of the literature

**DOI:** 10.1186/s12889-023-15875-6

**Published:** 2023-05-23

**Authors:** Kristine D. Gu, Katherine C. Faulkner, Anne N. Thorndike

**Affiliations:** 1 Division of Endocrinology, Massachusetts General Hospital, 50 Staniford Street, Suite 340, Boston, MA 02114 USA; 2 Harvard Medical School, Boston, MA USA; 3 Division of General Internal Medicine, Massachusetts General Hospital, Boston, MA USA

**Keywords:** Housing instability, Cardiometabolic health, Overweight, Obesity, Hypertension, Diabetes, Cardiovascular disease

## Abstract

Housing instability is variably defined but generally encompasses difficulty paying rent, living in poor or overcrowded conditions, moving frequently, or spending the majority of household income on housing costs. While there is strong evidence that people experiencing homelessness (i.e., lack of regular housing) are at increased risk for cardiovascular disease, obesity, and diabetes, less is known about housing instability and health. We synthesized evidence from 42 original research studies conducted in the United States examining the association of housing instability and cardiometabolic health conditions of overweight/obesity, hypertension, diabetes, and cardiovascular disease. The included studies varied widely in their definitions and methods of measuring housing instability, but all exposure variables were related to housing cost burden, frequency of moves, living in poor or overcrowded conditions, or experiencing eviction or foreclosure, measured at either the individual household level or at a population level. We also included studies examining the impact of receipt of government rental assistance, which serves as a marker of housing instability given that its purpose is to provide affordable housing for low-income households. Overall, we found mixed but generally adverse associations between housing instability and cardiometabolic health, including higher prevalence of overweight/obesity, hypertension, diabetes, and cardiovascular disease; worse hypertension and diabetes control; and higher acute health care utilization among those with diabetes and cardiovascular disease. We propose a conceptual framework for pathways linking housing instability and cardiometabolic disease that could be targeted in future research and housing policies or programs.

## Background

Housing instability is variably defined but generally encompasses difficulty paying rent, living in overcrowded conditions, moving frequently, spending the majority of household income on housing costs [1], or experiencing poor housing quality or unstable neighborhood environments [2, 3]. It has been associated with decreased access to routine healthcare, increased acute care utilization, and poor self-rated and mental health [2, 4–7], and is widely considered to be a fundamental social determinant of health. Housing instability disproportionately affects racial and ethnic minorities whose housing and economic opportunities have been restricted due to a history of discriminatory housing practices in the United States [8]. Rooted in structural racism, these practices forced minority groups such as Blacks and Hispanics into disadvantaged housing [9] and perpetuate residential racial segregation, which has been associated with poor health outcomes [10]. Housing instability is also a risk factor for homelessness, defined as lacking a regular nighttime residence or having a primary nighttime residence that is a temporary shelter or place not designed for sleeping [11]. While there is strong evidence that people experiencing homelessness have high rates of overweight and obesity [12, 13], barriers to diabetes management and health care access [14], increased risk for uncontrolled diabetes [15], and higher cardiovascular disease morbidity and mortality [16], less is known about the impact of housing instability on cardiometabolic health. Although housing instability represents a less severe housing status compared to homelessness, its implications on both the health of individuals and families, as well as on racial and ethnic health disparities, have increasingly become a focus of public health research and policy efforts [8, 10].

**Fig. 1:**
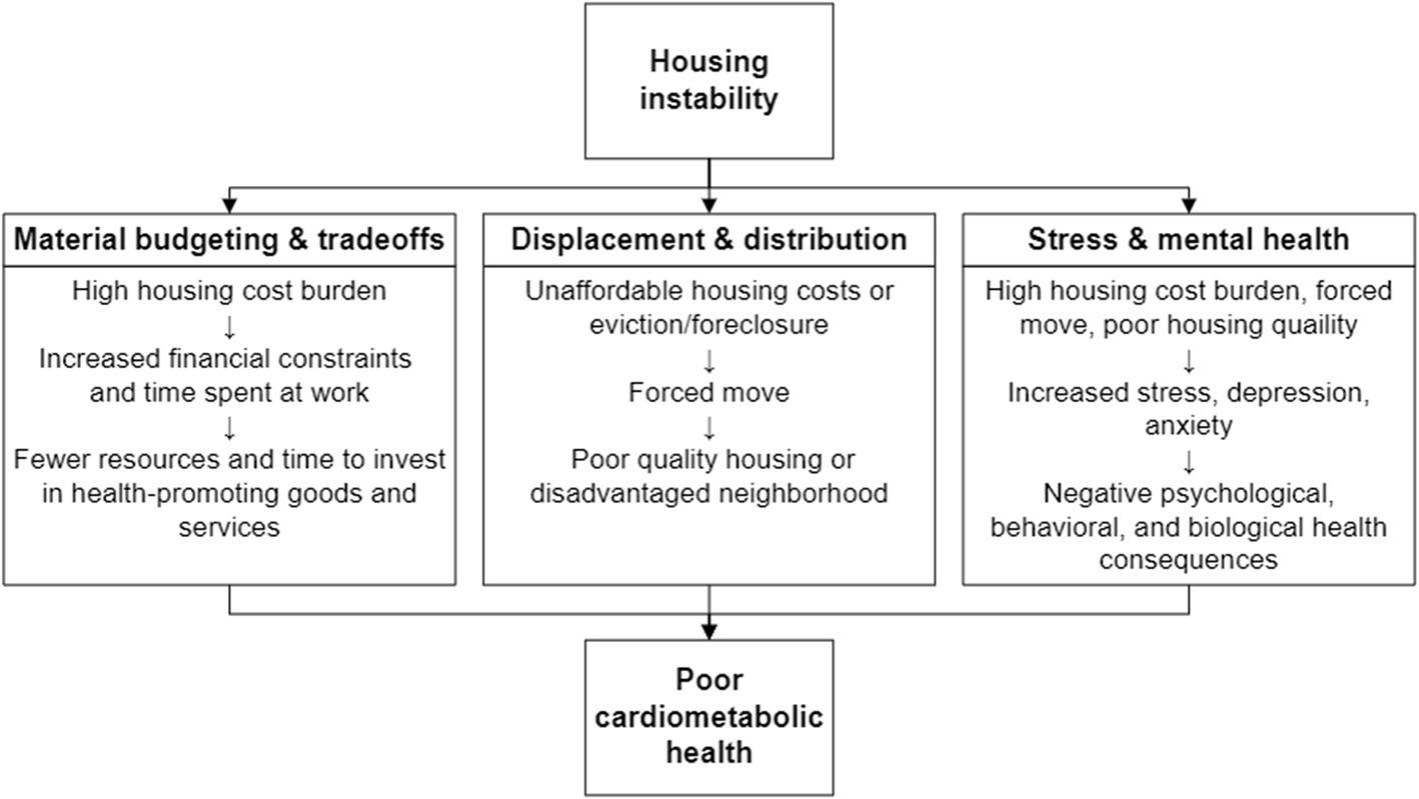
Proposed pathways linking housing instability and cardiometabolic health (adapted from Downing and Rodgers et al. [17, 18])

Several mechanisms linking housing affordability and both physical and mental health have been proposed in a recent systematic review by Downing and expanded upon by Rodgers et al. [17, 18]. We draw upon three of these proposed mechanisms to help explain how housing instability in general may impact cardiometabolic health: 1) material budgeting and tradeoffs, 2) displacement and distribution into disadvantaged environments, and 3) psychosocial stress and mental health (Fig. 1). The first mechanism, material budgeting and trade-offs, occurs in response to high housing cost burden, as decreased financial resources lead to lower capacity to purchase or access health-promoting goods and services, such as healthy foods, medications, and healthcare. Financial restraints may also cause individuals to work longer hours or additional jobs, leaving less time for health-promoting behaviors, such as physical activity, sleep, or medical appointments [17, 18]. In the second pathway of displacement and distribution, unaffordable housing costs and evictions or foreclosures can lead to forced moves, displacing households and distributing them into disadvantaged neighborhoods with multiple factors that can harm health, such as poor housing quality, environmental detriments like crime, pollution, and toxins, or decreased availability of healthy foods or safe recreation areas [17–19]. Finally, high housing cost burden, forced moves, or the perception of poor housing quality can lead to psychosocial stress, depression, and anxiety [20], which have been linked to obesity [21–24], cardiovascular disease [25–28], and diabetes [25, 29, 30].

An emerging body of literature has examined associations between cardiometabolic health outcomes and various dimensions of housing instability. Due to a lack of a singular definition, measures of housing instability vary widely in the literature [2, 20, 31]. Housing stability is often considered to represent one of four core dimensions of housing, along with affordability, quality, and neighborhood environment, each representing a pathway by which housing affects health [32–34]. While these pathways are often distinctly delineated in the literature, there is significant overlap of each of these dimensions of the housing construct. In this review, we broadly define the term housing instability, which is often used interchangeably with the term housing insecurity, to include problems with housing stability, affordability, and quality. Table 1 provides a summary of definitions and descriptions of the various components that can be used as measures of housing instability, including degree of difficulty affording housing costs (i.e., housing cost burden), frequency of moves, forced moves due to eviction or foreclosure, living in overcrowded conditions, living with friends or relatives to spilt housing costs (i.e., doubling up [35]), or experiencing poor housing quality. Use of government rental assistance programs may also be considered an indicator of housing instability, given that the goal of these programs is to alleviate housing cost burden for low-income households. Additionally, each of these components can represent exposure variables assessed at the individual household level, or aggregated by populations located within a geographical area, such as a county or census block [17]. Individual-level studies have examined the effects of direct household exposure to housing instability using participant surveys, interviews, or other screening tools. Population-level studies of housing instability have assessed the impacts of overcrowding, housing cost burden, or eviction and foreclosure rates affecting populations located within geographical area, such as census-level foreclosure rates or proportion of total household income spent on housing costs, aggregated to the county level.

**Table 1:** Definitions and descriptions of terms and policies related to housing instability and government rental assistance

Housing Term/Policy	Definition/Description
Housing affordability	Generally defined as housing for which the occupant is paying no more than 30% of gross income for housing costs, including utilities [11]
Housing cost burden	The experience of spending more than 30% of household income on housing costs. Severe housing cost burden refers to the experience of spending more than 50% of income on housing costs [1, 36]
Overcrowding	Commonly defined as the presence of > 1 person per room or > 2 people per bedroom living within a housing unit [37]
Doubling up	Living with one or more adults in addition to the head of household and their spouse or partner to share living expenses, such as an adult child living at home for financial reasons, two related or unrelated families residing together, or a parent living with an adult child [35, 38]
Poor housing quality	Inadequate or unsafe physical conditions of a place of residence, such as the presence of lead, mold, or asbestos, poor air quality, poor thermal regulation, or overcrowding, in the home [39]
Eviction	An involuntary move of a tenant from a leased unit as a result of landlord-initiated termination of lease [11]
Foreclosure	Legal process by which a property may be sold with proceeds applied to the mortgage debt when the loan becomes delinquent because payments have not been made or when the homeowner is in default for a reason other than the failure to make timely mortgage payments [40]. Also known as mortgage possession
Forced move or displacement	Removal from one’s home as a result of eviction or foreclosure [1]
Government rental assistance or subsidized housing	Generic term to describe any federal, state, or local governmental program that reduces the cost of housing for low- or moderate-income households; includes tenant-based or unit-based housing assistance programs [41]
Tenant-based housing assistance (vouchers)	Subsidies to help low-income households (that make less than 50% of the median household income in a particular county or metropolitan area) rent housing in the private market; currently termed housing choice vouchers by HUD but previously termed Sect. 8 certificates or vouchers. Tenant pays 30% of income towards a payment standard set by PHAs to represent the amount generally needed to rent a moderately-priced housing unit in the local private market, with the remainder subsidized by HUD. Moves are permissible and subsidies follow tenants between homes under certain regulations [42, 43]
Unit-based housing assistance	HUD provides subsidies to PHAs (public housing) or private landlords (project-based rental assistance) to provide affordable housing to low-income tenants. Rent subsidy is tied to the unit and does not follow tenants after they leave. Also called project-based subsidies or project-based vouchers [43–46]

*Legend*: *HUD* United States Department of Housing and Urban Development, *PHA* Public housing agency

The purpose of this narrative review is to summarize the literature on the relationship between housing instability and cardiometabolic health. We synthesized evidence from 42 original research studies conducted in the United States examining the relationship between various individual- or population-level exposures of housing instability, including housing cost burden, frequent moves, overcrowding/doubling up, poor housing quality, rental assistance use, and foreclosures and evictions, and the cardiometabolic conditions of overweight/obesity, hypertension, diabetes, and cardiovascular disease in adult populations.

## Methods

This review includes original research studies examining the relationship between housing instability and cardiometabolic health in US adult populations collected through a search of Pubmed and Scopus databases. Search terms included “cardiometabolic risk,” “overweight and obesity,” “hypertension,” diabetes and prediabetes,” “coronary disease,” “heart failure,” and “stroke,” in combination with “housing instability,” “housing insecurity,” “unstable housing,” “housing affordability,” “housing quality,” “housing conditions,” “foreclosures or mortgage possessions,” “evictions,” “rental assistance,” “housing assistance,” and “public housing.” These search terms yielded 394 English-language abstracts from the databases. We excluded animal studies, review articles, book chapters, editorials, commentaries, studies that focused on children, and literature examining the association between the built or neighborhood environment and cardiometabolic health, which has been comprehensively reviewed in prior literature [47–52]. We also excluded studies that focused exclusively on people experiencing homelessness as this area has been widely studied and reviewed and may have different implications for health outcomes and management of disease [35]. One hundred fifty abstracts remained after applying our exclusion criteria.

We reviewed these 150 abstracts to isolate studies examining the association between cardiometabolic health and the exposure variable of housing instability in the form of housing cost burden, frequent moves, overcrowding/doubling up, poor housing quality, or foreclosures or eviction. We also included studies exploring the exposure to government rental assistance programs, such as tenant-based (i.e., vouchers) and unit-based (i.e., public housing or project-based assistance) rent subsidies, as potential markers of current or recent housing instability. Forty articles met our inclusion criteria, with the remaining articles excluded as they did not specifically examine associations between housing instability as the exposure variable and the cardiometabolic conditions or outcomes of interest. While review articles were excluded from the results of our paper, we identified one additional article [53] absent from the database search after reading a pertinent review [43], and one article was identified during the peer review process [54]. We then grouped studies by cardiometabolic condition (overweight and obesity, hypertension, diabetes, and cardiovascular disease), and further categorized them based on whether the housing instability exposure represented an individual or population-level variable. Selected characteristics and key findings of the 42 included studies are provided in Table 2.

**Table 2:** Selected characteristics of included studies

Author, year	Study design	Setting	Sample	Measure(s) of housing instability exposure	Cardio- metabolic health condition	Relevant health outcome(s) examined	Key findings
**Individual-level housing instability exposure**							
Antonakos & Colabianchi, 2018 [55]	Cohort	Nationally representative survey (PSID)	1374 adults not receiving rental assistance at baseline: 116 were receiving assistance at year 2 (treatment group); 1258 eligible for but not receiving assistance at year 2 (control group)	Rental assistance: public housing or other project-based housing (including low-income tax credit), tenant-based housing (i.e., housing vouchers), and state-assisted housing	Obesity	BMI, obesity prevalence, physical activity	Any type of rental assistance use at 2-year follow-up associated with higher obesity at 2-year follow-up (coef. 0.14, SE 0.07; *p* = 0.04) compared to those eligible for but not receiving rental assistance (excluded permanently disabled people)
Berkowitz et al. 2015 [56]	Cross-sectional	Massachusetts	411 adults with diabetes from a practice-based research network (2 CHCs, 1 academic general internal medicine clinic, 1 specialty diabetes clinic)	Housing instability: evictions, frequent moves, doubling up, or homelessness	Diabetes	Diabetes control (composite measure of HbA1c > 9% (74.9 mmol/mol), LDL > 100 mg/dL (2.6 mmol/L), BP > 140/90 mmHg); outpatient and acute care (ED and inpatient) utilization	Housing instability associated with higher likelihood of poor diabetes control (*p* = 0.04) and outpatient visits (*p* = 0.03) in unadjusted analyses; after adjustment for covariates only association with outpatient visits remained statistically significant (IRR 1.31, 95% CI 1.14, 1.51)
Berkowitz et al. 2018 [35]	Cross-sectional	Nationally representative survey (HCPS)	1,087 adults with diabetes receiving care at safety-net health centers	Unstable housing: not having enough money to pay rent/mortgage, moving ≥ 2 times in the past 12 months, or doubling up	Diabetes	Diabetes-associated ED or inpatient healthcare utilization (self-reported)	Unstable housing associated with higher odds of diabetes-related ED or inpatient use (adjusted OR 5.17, 95% CI 2.08, 12.87)
Beverly et al. 2020 [57]	Qualitative interviews	Southeastern Ohio	42 providers who treat people with diabetes	Housing insecurity	Diabetes	Participants’ perspectives on diabetes management	Housing insecurity identified as a significant issue complicating diabetes management
Chambers & Rosenbaum, 2014 [58]	Cross-sectional	Bronx, New York	371 Hispanic/Latino adults from randomly selected households	Rental assistance: public housing, Sect. 8 vouchers	CVD	CVD-related outcomes: history of heart attack or stroke (self-reported), hypertension	Lower odds of CVD-related outcomes in unassisted housing group (OR 0.394, 95% CI 0.204, 0.761, p ≤ 0.01) and housing voucher group (OR 0.527, 95% CI 0.280, 0.992, p ≤ 0.05) compared to public housing group
Chambers et al. 2021 [59]	Cross-sectional	Bronx, New York	5846 adults with type 2 diabetes receiving care at Bronx, New York-based hospital system	Social needs: housing quality, housing instability (worry about eviction/ homelessness), food insecurity, health transportation, cost-related healthcare nonadherence, utility insecurity, conflicts with family, child or elderly care needs, legal needs, and intimate partner violence	Diabetes	Diabetes control (HbA1c)	Presence of ≥ 3 social needs associated with higher likelihood of uncontrolled diabetes defined as HbA1c ≥ 9.0% (74.9 mmol/mol, adjusted OR 1.59, 95% CI 1.26, 2.00. Presence of housing issues (including needs related to housing quality and instability) associated with higher likelihood of uncontrolled diabetes (*p* < 0.05)
Charkhchi et al. 2018 [60]	Cross-sectional	Nationwide survey (BRFSS)	84,353 US adults	Housing insecurity: worry about paying rent/mortgage	CVD	CVD: myocardial infarction, angina, coronary heart disease (self-reported)	CVD associated with increased odds of housing insecurity (OR 1.69, 95% CI 1.07, 2.66)
Fenelon et al. 2022 [61]	Cohort	Nationally representative survey (NHANES)	1050 adults receiving rental assistance or on a waitlist at time of NHANES survey	Rental assistance: project-based housing, housing vouchers	Diabetes	HbA1c	Project-based housing associated with decreased prevalence of HbA1c ≥ 9.0% (74.9 mmol/mol; prevalence − 3.7%, 95% CI − 7.0, 0.0%) compared to waitlist (prevalence 6.9%); no significant differences in HbA1c between voucher and waitlist groups
Fertig & Reingold, 2007 [62]	Cohort	Nationally representative survey (FFCWS)	3 subsamples: 1) 422 mothers living in public housing at baseline vs. 2055 in comparison group; 2) 323 mothers who moved into public housing between baseline and year 1 vs. 1999 in comparison group; 3) 150 mothers with 2–3 children who moved into public housing between baseline and year 1 vs. 919 in comparison group (comparison groups eligible for but not living in public housing at each time point)	Rental assistance: public housing	Obesity	BMI	Mothers moving into public housing between baseline and 1-year follow-up more likely to be overweight at 3-year follow-up compared to mothers eligible for but not yet living in public housing (*p* = 0.05)
Fitzpatrick et al. 2021 [63]	Cross-sectional	Oregon and Southwest Washington	4043 insured adults from the Kaiser Permanente Northwest diabetes registry	Unmet basic needs: housing situation and concerns about housing (cost burden, stability, safety), food insecurity, financial hardship, transportation, help with ADLs	Diabetes	Diabetes control (HbA1c) and healthcare utilization (outpatient, ED, hospitalization, diabetes-related prescription refills)	Presence of one or more unmet basic needs associated with increased odds of HbA1c > 8% (63.9 mmol/mol), more outpatient and ED visits, and more delayed refills of diabetes medications compared to having no needs
Gaston et al. 2018 [64]	Cross-sectional	Nationally Representative Survey (NHIS)	80,880 non-Hispanic White and Black adult renters who were born in the US	Rental assistance: any type	Obesity, hypertension, diabetes, CVD	Hypertension, diabetes, heart disease, stroke, weight/height (self-reported)	Disparities in overweight/obesity, hypertension, and diabetes in Blacks with worse sleep compared to Whites with recommended sleep were generally smaller among government-assisted renters, but relationships varied by sex
Gold et al. 2022 [65]	Cross-sectional	186 CHCs with shared EHR across the US	73,484 adults with type 2 diabetes	Housing insecurity: EHR screening tool	Diabetes	Diabetic care quality (statin prescription; up-to-date HbA1c, microalbuminuria and LDL measurements; diabetic foot screening)	No difference in overall care quality for those with housing insecurity, except decreased likelihood of up-to-date LDL screening
Heller et al. 2021 [54]	Cross-sectional	Bronx and Westchester Counties, New York	33,550 adults receiving primary care at an academic medical center in New York	Social needs: housing quality, housing instability (worry about eviction/ homelessness), food insecurity, health transportation, cost-related healthcare nonadherence, utility insecurity, conflicts with family, child or elderly care needs, legal needs, and intimate partner violence	Obesity, hypertension, diabetes	Obesity, hypertension, and diabetes prevalence	Presence of 3 social needs associated with higher prevalence of obesity (PR 1.06, 95% CI 1.00–1.12), hypertension (PR 1.15, 95% CI 1.09–1.21), and diabetes (p-trend < 0.001) compared to no needs
Joynt Maddox et al. 2019 [66]	Cross-sectional	US, Medicare RIFs and vital records	2,952,605 Medicare patients hospitalized for acute MI, CHF, or pneumonia	Housing instability: ≥ 2 unique residential addresses in EHR claims data	CVD	Acute MI or CHF hospitalization	Medicare beneficiaries with housing instability hospitalized for acute MI or CHF had higher odds of 30-day hospital readmission compared those with stable housing
Kalousova & Evangelist, 2019 [53]	Natural experiment	Detroit, Michigan	2 subsamples: 1) 62 receiving rental assistance, 200 eligible but not receiving assistance, 100 ineligible at baseline; 2) 31 receiving assistance, 76 eligible but not receiving assistance, 43 ineligible at all exam waves	Rental assistance: any type	Obesity	BMI	No significant difference in BMI between adults receiving rental assistance compared to those eligible for but not receiving assistance at baseline or at 4-year follow-up
Keene, Guo et al. 2018 [67]	Qualitative interviews	New Haven, Connecticut	40 low-income adults with type 2 diabetes who either resided in or qualified for subsidized housing	Rental assistance: project-based housing, tenant-based vouchers	Diabetes	Participants' perspectives on how access to subsidized housing affected diabetes self-management	Access to subsidized housing affected participants' ability to prioritize diabetes care, establish and maintain diabetes routines, and afford diabetes-related expenses
Keene, Henry et al., 2018 [68]	Qualitative interviews	New Haven, Connecticut	23 low-income adults with type 2 diabetes who either resided in or qualified for subsidized housing	Transition into rent-assisted housing	Diabetes	Ability to manage diabetes care and experiences	Receipt of rental assistance was accompanied with improvements in diabetes self-management by promoting diabetes routines, freeing financial resources to pay for diabetes-related expenses, and mitigating financial stress
Leung et al. 2022 [69]	Cross-sectional	Michigan, University of Michigan EHR and Diabetes Research Registry	571 adults with type 1 or 2 diabetes, HbA1c ≥ 7.5% (58.5 mmol/mol), positive report of financial burden or cost-related non-adherence, and access to a mobile phone	Housing insecurity: worry about paying housing costs (exposure variable was cumulative social risk factor score which also included food, financial, and utility insecurity)	Diabetes	HbA1c, cost-related non-adherence (includes medication and health care), diabetes distress	Having 3–4 social risks associated with higher likelihood of cost-related non-adherence (OR 2.81, 95% CI 1.95, 4.06, *p* < 0.0001), diabetes distress (OR 3.03, 95% CI 2.13, 4.31, *p* < 0.0001), but only marginally higher mean HbA1c (β 0.36, 95% CI − 0.01, 0.72; *p* = 0.08), compared to those with no social risks
Lim et al. 2020 [70]	Natural experiment	New York City, New York	1460 adults without diabetes at baseline followed over 12 years: 730 residents of New York City Housing Authority (public housing) vs. 730 propensity score-matched adults not living in public housing	Housing stability: address changes over 12-year period; rental assistance: public housing	Diabetes	Diabetes incidence (self-reported)	New York City public housing residence was associated with higher prevalence of housing stability (PR 1.16, 95% CI 1.07, 1.25) but not reduced diabetes risk (RR 1.11, 95% CI 0.83, 1.48); among those with housing instability, public housing associated with increased diabetes risk (RR 1.59, 95% CI 1.01, 2.5)
Ludwig et al. 2011 [71]	Natural experiment	Five US cities: Baltimore, Maryland; Boston, Massachusetts; Chicago, Illinois; Los Angeles, California; and New York City, New York	4498 families from the MTO project, living in public housing located in high-poverty census tracts at baseline, randomized to 3 groups to receive: 1) housing vouchers redeemable for private-market housing located in low-poverty census tracts, and counseling to help with housing search; 2) standard housing vouchers with no restriction on where they could be redeemed; 3) no new housing assistance (control)	Housing quality: low-poverty voucher group given the opportunity to improve housing quality by moving to neighborhood with low poverty	Obesity, diabetes	BMI, HbA1c	Low-poverty voucher group had lower prevalence of BMI > 35 kg/m^2^ (absolute difference − 4.61%, 95% CI − 8.54, − 0.69), BMI > 40 kg/m^2^ (− 3.38, 95% CI − 6.39, − 0.36), and HbA1c ≥ 6.5% (47.5 mmol/mol) (− 4.31, 95% CI − 7.82, − 0.80) compared to control group. No differences between standard voucher and control groups
Mosley-Johnson et al. 2022 [72]	Cross-sectional	Nationally representative survey (BRFSS)	16,091 US adults	Housing insecurity: worry about paying housing costs	Diabetes	Diabetes processes of care (physician visits, HbA1c tests, dilated eye exams, diabetes education assessment, flu shot) and self-care behaviors (home blood glucose monitoring, home foot examination, physical activity)	Housing insecurity associated with decreased likelihood of having a physician visit (adjusted OR 0.58, 95% CI 0.37, 0.92), HbA1c assessment (adjusted OR 0.45, 95% CI 0.26, 0.78), or eye exam (adjusted OR 0.61, 95% CI 0.44, 0.83) in employed adults, and decreased likelihood of flu vaccine (adjusted OR 0.84, 95% CI 0.70, 0.99) in unemployed adults
Mulugeta et al. 2021 [73]	Cohort	Massachusetts	11,534 non-pregnant adults receiving care at a large safety-net health system	Housing insecurity: EHR screening tool (exposure variable was combined with food insecurity)	Obesity	BMI	Men with food or housing insecurity had higher odds of ≥ 5% weight gain (OR 1.44, 95% CI 1.05, 1.97)
Pollack et al. 2010 [5]	Cross-sectional	Pennsylvania (Southeastern Pennsylvania Household Health Survey)	10,004 adult residents of Philadelphia County and 4 surrounding counties	Housing affordability: degree of difficulty affording housing costs	Hypertension, diabetes, CVD, Obesity	Hypertension, diabetes, cardiovascular disease (self-reported); cost-related non-adherence (medication and health care); ED use; BMI	Unaffordable housing associated with increased odds of poor self-rated health (adjusted OR 1.75, 95% CI 1.33, 2.29), hypertension (adjusted OR 1.34, 95% CI 1.07, 1.69), cost-related health care non-adherence (adjusted OR 2.94, 95% CI 2.04, 4.25), and cost-related prescription nonadherence (adjusted OR 2.68, 95% CI 1.95, 3.70). No significant associations between housing affordability and diabetes, CVD, or obesity
Pollack et al. 2011 [74]	Case–control	Pennsylvania (University of Pennsylvania Hospital System EHR)	404 adults who received home foreclosure notice (cases) vs. 2020 controls residing in same ZIP code as cases	Receipt of foreclosure notice	Hypertension, diabetes, CVD	Hypertension, renal disease, diabetes, heart disease diagnoses (EHR diagnosis)	People undergoing foreclosure had higher prevalence of hypertension (adjusted OR 1.40, 95%, CI 1.08, 1.81; *p* = 0.01) and renal disease (adjusted OR 1.83, 95% CI 1.09, 3.06; *p* = 0.02). Differences in cerebrovascular disease marginally but not significantly higher (adjusted OR 1.56, 95% CI 0.97, 2.52; *p* = 0.07). No significant differences in diabetes or heart disease
Schootman et al. 2007 [75]	Cohort	St. Louis, Missouri	998 African American adults living in St. Louis or surrounding suburbs	Housing quality: cleanliness inside building, physical condition of interior, condition of furnishings, condition of exterior of building, and global rating (excellent, good, fair, or poor)	Diabetes	Diabetes incidence (self-reported)	Fair/poor rating for each housing condition associated with increased risk of incident diabetes compared to good/excellent rating (cleanliness inside building OR 1.78, 95% CI 1.03, 3.07; physical condition inside building, OR 2.53, 95% CI 1.47, 4.34; condition of furnishings inside building, OR 2.20, 95% CI 1.29, 3.75; condition of the outside of the building, OR 2.39, 95% CI 1.40, 4.08; overall condition of the dwelling, OR 1.78, 95% CI 1.02, 3.09)
Stupplebeen, 2019 [76]	Cross-sectional	Hawaii (BRFSS)	9907 White, Asian, and NHOPI adults	Housing insecurity: worry about paying rent/mortgage	Diabetes, CVD	Diabetes (self-reported), CVD (self-reported coronary heart disease or stroke)	Housing insecure NHOPIs had higher odds of diabetes (OR 1.85, 95% CI 1.13, 3.01) and CVD (OR 1.85, 95% CI 1.04, 3.28) compared to housing secure NHOPIs
Thomas et al. 2022 [77]	Cohort	Northern California	17,317 continuously insured adults from the Kaiser Permanente Northern California diabetes registry	Housing insecurity: at least one address change in EHR	Diabetes	BP, HbA1c, ED visits, flu vaccine receipt	≥ 1 address change associated with greater chance of HbA1c > 9% (74.9 mmol/mol; ARR 1.12, 95% CI 1.09, 1.15), lower chance of HbA1c < 8% (63.9 mmol/mol; ARR 0.95, 95% CI 0.94, 0.96), lower chance of controlled BP (ARR 0.99, 95% CI 0.98, 0.99), higher chance of ED Visit (ARR 1.25, 95% CI 1.23, 1.27, lower chance of flu vaccine (ARR 0.94, 95% CI 0.93, 0.95) when compared to no address change
Vijayaraghavan et al. 2011 [78]	Cross-sectional	San Francisco, CA and Chicago, Illinois (ICHC study)	711 Mexican American, African American, and non-Hispanic White adults with diabetes receiving care at safety net practices	Housing instability: ordered into 5 categories of 1) owning home, condominium, or apartment, 2) living with family, 3) renting an apartment, 4) renting a room in another person's home, or 5) lacking a usual place to live (could include homelessness)	Diabetes	Diabetes care self-efficacy	Increase in housing instability associated with linear decrease in diabetes self-efficacy (*p* < .01) in unadjusted models. In adjusted models, adults without usual place to stay had lower self-efficacy than those who owned their own home (β − 0.94, 95% CI − 1.88, − 0.01)
Vijayaraghavan et al. 2013 [79]	Cohort	Four US cities (Birmingham, Alabama; Chicago, Illinois; Minneapolis, Minnesota; and Oakland, California)	4,342 Black and White adults followed over 15 years	Housing instability: frequent moves, housing crowding, and occupying a place without rent or money	Hypertension	Hypertension incidence (SBP ≥ 140 mmHg, DBP ≥ 90 mmHg, or self-reported anti-hypertensive medication use)	Housing instability was not associated with incident hypertension (IRR 1.1; 95% CI, 0.9–1.5), but did vary by race and sex (*p* value for interaction, < 0.001), with unstably housed white women having higher risk of hypertension (IRR 4.7, 95% CI 2.4, 9.2)
**Population-level housing instability exposures**							
**Author, year**	**Study design**	**Setting**	**Sample**	**Measure(s) of housing instability exposure**	**Cardio- metabolic health condition**	**Relevant health outcome(s) examined**	**Key findings**
Arcaya et al. 2013 [80]	Cohort	Massachusetts (Framingham Offspring Cohort)	2068 adults from the Framingham Offspring Cohort followed over 5 exam waves between 1987–2008	Number of proximate foreclosures within 100 m of person's home in 1 year leading up to study visit	Obesity	BMI, odds of overweight	Each additional foreclosure associated with 0.20 units increase in BMI (95% CI 0.03, 0.36), and increased risk of being overweight (OR 1.77 (95% CI 1.02, 3.05)
Arcaya et al. 2014 [81]	Cohort	Massachusetts (Framingham Offspring Cohort)	1740 adults from the Framingham Offspring Cohort followed over 5 exam waves between 1987–2008	Number of proximate foreclosures within 100 m of person's home in 1 year leading up to study visit	Hypertension	SBP ≥ 140 mmHg, DBP ≥ 90 mmHg, or on antihypertensive treatment	Each additional foreclosure associated with increase in SBP of 1.71 mmHg (95% CI 0.18, 3.24; *p* = 0.03)
Chambers et al. 2019 [82]	Cross-sectional	Four US cities: Chicago, Illinois; Miami, Florida; Bronx, New York; San Diego, California	13,856 low-income Latino adults residing in public housing units, housing subsidized by Sect. 8 vouchers, and housing with no federal assistance	Foreclosure risk estimated by HUD as a function of area decline in home values, unemployment rate, and high-cost mortgage loans	Hypertension	SBP ≥ 140 mmHg, DBP ≥ 90 mmHg, and/or receiving antihypertensive medication, total cholesterol ≥ 240 mg/dL (6.2 mmol/L), LDL cholesterol ≥ 160 mg/dL (4.1 mmol/L), or HDL cholesterol < 40 mg/dL (1.0 mmol/L) or receiving cholesterol-lowering medications	Living in a high foreclosure risk area associated with higher prevalence of hypertension (PR 1.25, 95% CI 1.08, 1.46) and hypercholesterolemia (PR 1.12, 95% CI 1.01, 1.24) compared to medium or low foreclosure risk areas
Christine et al. 2017 [83]	Cohort	6 US cities: New York City, New York; Baltimore, Maryland; Forsyth County, North Carolina; Chicago, Illinois; St. Paul, Minnesota; and Los Angeles, California	3775 White, Black, Hispanic, and Chinese adults without baseline cardiovascular disease	Change in number of foreclosures filings within a quarter mile of participant’s residence between examination years	Diabetes, hypertension, obesity	BMI, SBP, and fasting glucose levels measured at study examinations	Increase of 1.9 neighborhood foreclosures per quarter mile associated with increase in fasting glucose (mean 0.22 mg/dL, 95% CI − 0.05, 0.50) and decrease in SBP (mean − 0.27 mmHg, 95% CI − 0.49, − 0.04) at follow-up exam (mean 4.61 years); no association between change in neighborhood foreclosure rate and BMI
Downing et al. 2016 [84]	Cohort	Northern California	105,919 insured adults with diabetes receiving care at Kaiser Permanente Northern California	Annual foreclosures rate per census block	Obesity	BMI (annual average over 4-year period)	No association between neighborhood foreclosure rate and change in BMI
Downing et al. 2017 [85]	Cohort	Northern California, insured population	105,930 insured adults from the Kaiser Permanente Northern California diabetes registry	Annual foreclosures rate per census block	Diabetes	HbA1c (annual average over 4-year period); diabetes control (1 measurement of HbA1c ≥ 9% [74.9 mmol/mol])	No association between neighborhood foreclosure rate and change in HbA1c
Duran et al. 2019 [86]	Cohort	6 counties in Chicago metropolitan area	59,854 adults receiving primary care at VA facility	Number of foreclosure filings within 100, 200, 500, or 1,000 m of individual’s home location	Obesity	BMI	No association between neighborhood foreclosures and BMI in overall sample; in a sample restricted to non-movers, every 20 additional foreclosures was associated with 0.03 kg/m^2^ increase in BMI (95% CI 0.01, 0.06)
Hazekamp et al. 2021 [87]	Cross-sectional	Urban Illinois	1267 census tracts	Annual eviction and eviction filing rates	Obesity	Obesity prevalence (from CDC's 500 Cities database)	Census-level eviction and eviction filing rates associated with obesity prevalence (β 2.203, 95% CI 1.970, 2.436)
Hohl and Lotfata, 2022 [88]	Cross-sectional	Chicago, Illinois	796 census tracts	Severe rent (% of population spending > 50% of income on rent) at census-level	Obesity	Obesity prevalence (from CDC's PLACES project database)	Obesity is positively associated with census-level severe rent (coef. 0.37, *p* < 0.01), and the area of Chicago with highest obesity also had the highest severe rent problem
Jones et al. 2020 [89]	Cross-sectional	US, SMART project (uses BRFSS data)	75 of the 100 most populous US metropolitan areas	Foreclosures: rate of mortgage possessions	Obesity	BMI	One unit increase in the 2009 foreclosure rate associated with a 0.68-point increase (*p* < 0.001) or 0.57-point increase (*p* < 0.05) in 2010 obesity prevalence using two separate models which included different measures of racial residential segregation
Lotfata & Tomal 2022 [90]	Cross-sectional	Chicago, Illinois	793 census tracts	Severe rent (% of population spending > 50% of income on rent), eviction rates, and crowded housing (% of occupied housing units with ≥ 1 occupant per room) at census-level	Obesity	Obesity prevalence, BMI	Census-level severe rent and eviction rates were positively associated with obesity prevalence in nearly all census tracts in Chicago
Rodgers et al. 2019 [18]	Cohort	National representative survey (NLSY79)	3,722 adults without baseline hypertension, diabetes, or obesity	Housing affordability: proportion of total household income spent on housing costs, aggregated to county level	Obesity, hypertension, diabetes	Hypertension, diabetes (self-reported diagnosis), BMI, use of antihypertensives (in those with hypertension diagnosis)	1% increase of county-level household income spent on housing costs associated with increased obesity (OR 1.37, 95% CI 1.00, 1.87; *p* = 0.049) and hypertension incidence (OR 1.22, 95% CI 1.06, 1.42; *p* = 0.01). Among those with incident hypertension, increase in housing cost burden associated with lower likelihood of antihypertensive use (OR 0.79, 95% CI 0.65, 0.97; *p* = 0.03). No significant association for diabetes
Segar et al. 2022 [91]	Cross-sectional	US	301,500 adults from the AHA GWTG-HF registry hospitalized with HF as primary diagnosis	Housing instability: ZIP code-level % of population renting home, % housing vacancy, % mobile homes, % living in housing with 10 + occupants	CVD	LOS in days for HF hospitalization	Each % increase in population living in overcrowded housing was associated with longer LOS for both racial groups (Black: β 1.30, 95% CI 0.47, 2.13; *p* = 0.002; White: β 1.10, 95% CI 0.65, 1.55, *p* < 0.001)

*Legend*: *ADL* Activities of daily living, *AHA* American Heart Association, *ARR* Adjusted risk ratio, *BMI* Body mass index, *BRFSS* Behavioral Risk Factor Surveillance System, *CHC* Community health center, *CHF* Congestive heart failure, *COVID-19* Coronavirus disease 2019, *CVD* Cardiovascular disease, *HER* Electronic health record, *FFCWS* Fragile Families and Child Wellbeing Study, *GWTG-HF* Get With The Guidelines-Heart Failure, *HbA1c* Hemoglobin A1c, *HCPS* Health Center Patient Survey, *HF* Heart failure, *HUD* US Department of Housing and Urban Development, *ICHC* Immigration, Culture and Health Care, *IRR* Incidence rate ratio, *LDL* Low density lipoprotein, *LOS* Length of stay, *MI* Myocardial infarction, *MTO* Moving to Opportunity for Fair Housing Demonstration Program, *NHOPI* Native Hawaiian or other Pacific Islander, *NHANES* National Health and Nutrition Examination Survey, *NLSY79* National Longitudinal Survey of Youths 1979, *OR* Odds ratio, *PR* Prevalence ratio, *PSID* Panel Study of Income Dynamics, *RIF* Research identifiable file, *RR* Relative risk, *SE* Standard error, *SBP* Systolic blood pressure, *SMART* Selected Metropolitan/Micropolitan Area Risk Trends, *US* United States, *VA* Veterans Administration, *ZIP* Zone improvement plan

Throughout the literature, there was significant variability in the methods used to measure housing instability as well as the terminology used to refer to similar concepts (e.g., “housing instability,” “housing insecurity,” or “unstable housing”). In the results, we include the housing terms used in the original research articles and describe how the housing exposure was measured in the study. A few studies included homelessness in the broader definition of housing instability [56, 57, 78] but most studies reviewed did not explicitly include people experiencing homelessness in their sample populations. Regarding housing quality, we only include studies which assessed the effects of the perception of poor housing quality (e.g., poor housing quality reported on a study survey). The perception of or dissatisfaction with inadequate housing quality has been linked to a feeling of limited control over one’s housing circumstance [20], which may have important implications on health. We exclude the large body of literature demonstrating associations of specific household environmental toxins (e.g., lead, air pollutants) or adverse conditions (e.g., cold indoor temperature) with poor respiratory and cardiometabolic health, as these relationships have been comprehensively reviewed in prior literature [92–97].

## Results

### Overweight/obesity

#### Individual-level studies

Seven quantitative studies explored the relationship between housing instability on the individual level and weight status. While none examined housing instability alone as a primary exposure variable, two studies assessed housing instability in combination with other measures of social determinants of health to represent a composite exposure of social risk. In a longitudinal cohort study of 11,543 adults in Massachusetts receiving care at a large safety net health system, authors demonstrated that within 3 months of the coronavirus disease-2019 (COVID-19) lockdown, men with food or housing insecurity (collected by an unspecified electronic health record [EHR] screening tool) had higher odds of at least a 5% weight gain (odds ratio [OR] 1.44, 95% confidence interval [CI] 1.05, 1.97) compared to those without food or housing insecurity [73]. In a cross-sectional study of 33,550 adults receiving primary care at an academic medical center in New York, Heller et al. found that having three social needs (measured by a survey which included questions on housing quality and instability, as well as other social determinants of health such as food, utility, and transportation insecurity) was associated with higher prevalence of obesity (prevalence ratio [PR] 1.06, 95% CI 1.00–1.12) compared to having no needs [54].

Five studies explored the effect of government rental assistance on weight. A longitudinal cohort study by Fertig and Reingold using data from the Fragile Families and Child Wellbeing Study found that mothers moving into public housing between baseline and year one of follow-up had increased likelihood of being overweight at three-year follow-up compared to mothers eligible for but not yet living in public housing [62]. Another longitudinal cohort study of 116 adults receiving rental assistance, defined as use of public housing, other project-based housing including low-income housing tax credit, tenant-based housing (mostly vouchers), or state-assisted housing, between baseline and two-year follow-up, found moderate but not significant increases in body mass index (BMI) and obesity at two-year follow-up compared to the 1258 matched adults who were eligible for, but did not receive, rental assistance. In a sensitivity analysis excluding permanently disabled participants, authors found significantly higher obesity at two-year follow-up in the group receiving rental assistance, though this difference did not persist at four- or six-year follow-up [55]. Fertig and Reingold suggested that the increase in obesity associated with public housing residence may be due to factors in the neighborhood environment that promote weight gain, or due to increased financial resources created by housing subsidies that are then diverted to purchase of unhealthy food or excess calories [62].

The Moving to Opportunity (MTO) for Fair Housing Demonstration Program, a large, randomized housing mobility project by the US Department of Housing and Urban Development (HUD) intended to uncover the effects of neighborhood characteristics on social and health outcomes, showed similar associations with obesity. The MTO project randomized 4498 women with children living in public housing located in high-poverty census tracts of five large, urban US cities to one of three groups to receive: 1) housing vouchers usable for private-market housing located in low-poverty census tracts, and counseling to help with their housing search; 2) standard vouchers with no restrictions on where they could reside; and 3) no additional housing assistance (control). In an analysis of the MTO project, Ludwig et al. found that women from families in the low-poverty voucher group, who were given the opportunity to move out of public housing located in high-poverty census tracts into private-market housing located in low-poverty census tracts, had lower prevalence of BMI > 35 kg/m^2^ (− 4.61%, 95% CI − 8.54%, − 0.69%) and BMI > 40 kg/m^2^ (− 3.38%, 95% CI − 6.39%, − 0.36%) at a mean follow up of 12.6 years compared to the control group [71]. In contrast to these studies, a study by Kalousova using data from the Michigan Recession and Recovery Study found that there was no difference in BMI between adults receiving any type of rental assistance versus those eligible for but not receiving assistance [53].

In a study using repeated, cross-sectional data from the National Health Interview Survey to examine racial differences in sleep and cardiometabolic health by government-assisted rental housing status, Gaston et al. compared the prevalence of overweight/obesity in Black versus White adults by sleep duration category (i.e., short sleepers, recommended sleepers, and long sleepers). The study found that among government-assisted renters, there were no racial differences in overweight/obesity prevalence in men across sleep duration categories. However, among unassisted renters, Black male short and recommended sleepers had higher prevalence of overweight and obesity compared to White male recommended sleepers. In women, Black short and recommended sleepers had higher prevalence of overweight/obesity regardless of housing status, though racial differences were more pronounced among those living in unassisted housing compared to government-assisted housing. Gaston et al. concluded that government-assisted housing narrowed the weight disparities seen in Black men with worse sleep compared to White men with recommended sleep durations; however, racial disparities persisted in women regardless of housing tenure. The authors noted that since women are often primary caregivers of families, these gender differences highlight an area that deserves future research given its potential implications on maternal and child health [64].

#### Population-level studies

Nine quantitative studies examined the association between population-level measures of housing instability and weight. Three studies found that housing cost burden was associated with higher obesity prevalence. A study by Rodgers et al. examining the association between housing cost burden (measured as the proportion of total household income spent on housing costs aggregated to the county level) and cardiovascular disease risk factors found that a one percent increase in median county-level household income spent on housing costs was associated with a 37% increase in the odds of obesity. This association was stronger both in renters compared to homeowners, and in men compared to women, when results were stratified by housing tenure and gender, respectively. The authors proposed that the higher obesity risk in men may be due to increased susceptibility to financial stress, or lower healthcare utilization, compared to women [18]. Using geographically weighted regression (GWR) which allows for measurement in spatial variation of regression models, Hohl and Lotfata found that obesity was positively associated with severe rent (defined as percentage of population spending > 50% of income on housing rent) and that the region in Chicago with the worst severe rent problem also had the highest obesity prevalence using a spatiotemporal clustering technique [88]. Similarly Lotfata and Tomal used multiscale geographically weighted regression to find that severe rent and eviction rates are the main housing determinants associated with obesity prevalence in Chicago [90].

Six studies investigating the association between population-level eviction or foreclosure rates and weight produced mixed results. Hazekamp et al. found that the prevalence of obesity, as well as other unhealthy behavior indicators (i.e., binge drinking, current smoking status, lack of leisure-time physical activity, and short sleep), was associated with census-level eviction rates in urban Illinois communities [87]. Another cross-sectional study found that foreclosures in 75 of the top 100 most populous metropolitan areas in the United States were independently associated with obesity prevalence [89]. A longitudinal study followed 2068 adults from the Framingham Offspring Cohort over four examination waves between 1987–2008 to assess the association between area-level foreclosures and blood pressure. Authors found that each additional foreclosed property located within 100 m of a person’s home occurring in the year preceding the study examination was associated with a 0.2 kg/m^2^ increase in BMI and a 1.77 higher odds of being overweight (95% CI 1.02, 3.05) [80]. A cohort study of 59,854 adults receiving care at a Veterans Health Administration (VA) facility in metropolitan Chicago found no association between neighborhood foreclosures and BMI over six years of follow-up in the overall sample; however when restricting the sample to people who did not move over the study period, authors found that every 20 additional foreclosures was associated with a 0.03 kg/m^2^ increase in BMI (95% CI 0.01, 0.06) [86]. In contrast, a study of 105,919 continuously insured adults with diabetes in Northern California did not find an association between census-level foreclosure and BMI, though authors noted that the relatively shorter study period of four years may not have been long enough to detect an effect [84]. A longitudinal study by Christine et al. using data from the Multi-ethnic Study of Atherosclerosis (MESA) also found that there was no association between a standard deviation increase in neighborhood foreclosure count (1.9 foreclosures per quarter mile) and mean difference in BMI over a five-year follow-up period [83].

### Hypertension

#### Individual-level studies

Literature regarding the association of hypertension with individual-level housing instability, housing affordability, or foreclosures was limited to five quantitative studies. Results from three studies examining measures of housing instability alone or in combination with other social determinants of health generally showed higher incidence and prevalence of hypertension. One longitudinal study of 4,342 Black and White young adults participating in the Coronary Artery Risk Development In Young Adults (CARDIA) study found no association of housing instability (measured by interview questions regarding overcrowding, frequent moves, or occupying a space without paying rent) and incident hypertension over 15 years of follow-up in the overall sample. When stratified by race and sex, however, authors found that women with housing instability were at higher risk of incident hypertension (incidence rate ratio [IRR] 4.7, 95% CI 2.4, 9.2) compared to stably housed white women. The authors explained these differences could be attributed to uneven distribution of social and environmental risk factors [79]. A cross-sectional study of 10,007 individuals participating in biennial Southeastern Pennsylvania Household Health Survey explored the relationship between hypertension and housing affordability, assessed as the level of difficulty paying rent. Authors found that among homeowners and renters, difficulty paying rent was associated with increased odds of poor self-rated health (adjusted OR 1.75, 95% CI 1.33, 2.29) and hypertension (adjusted OR 1.34, 95% CI 1.07, 1.69). Authors suggested these findings were related to the knowledge that those with housing unaffordability have a higher likelihood of delaying or skipping doctors’ visits or accessing medications, and this was supported by their results demonstrating that high housing costs were associated with cost-related healthcare nonadherence (adjusted OR 2.94, 95% CI 2.04, 4.25) and cost-related prescription medication nonadherence (adjusted OR 2.68, 95% CI 1.95, 3.70) [5]. The previously mentioned cross-sectional study by Heller et al. examining housing instability and quality in combination with other social needs found that having three social needs was associated with higher prevalence of hypertension (PR 1.15, 95% CI 1.09, 1.21) compared to having no needs [54].

One study examined the relationship between hypertension prevalence and individual-level foreclosures, and another investigated the effect of government rental assistance on hypertension prevalence. A case–control study by Pollack et al. found significantly higher rates of hypertension (adjusted OR 1.40, 95% CI 1.08, 1.81) and renal disease (adjusted OR 1.83, 95% CI 1.09, 3.06) among 404 adult homeowners who received a foreclosure notice (cases) compared to the 2020 adults in the control group who received care from the hospital system and lived in the same zone improvement plan (ZIP) code as cases. Authors also found that people experiencing foreclosure were more likely to have an ED visit, outpatient visit, and no-show appointment, but less likely to have a PCP visit in the 6 months prior to foreclosure notice, suggesting that health care utilization patterns shift in the time period leading up to a foreclosure event [74]. In the previously cited study by Gaston et al. examining racial differences in sleep and cardiometabolic health by government-assisted rental housing status, among those in unassisted housing, Black male short and recommended sleepers had higher prevalence of hypertension compared to White recommended sleepers, a difference was not seen among those receiving government-assisted housing. In women, the prevalence of hypertension was higher in Blacks compared to Whites across all sleep categories and rental assistance categories, with larger differences seen among unassisted residents, highlighting the importance of future research on gender differences in racial/ethnic health disparities [64].

#### Population-level studies

Four quantitative population-based studies on the relationship between housing instability and hypertension produced mixed results. The study by Rodgers et al. cited previously found that each percentage point increase in county-level median percentage of household income spent on housing was associated with a 22% increase in the likelihood of incident hypertension (OR 1.22, 95% CI 1.06, 1.42) among renters and homeowners. When results were stratified by housing tenure and gender, this association was stronger in renters compared to homeowners, and in men compared to women, possibly owing to increased financial stressors and decreased healthcare use in men as previously mentioned. Additionally, among people with incident hypertension, a one unit increase in housing cost burden was associated with lower likelihood of antihypertensive medication use (OR 0.79, 95% CI 0.65, 0.97), suggesting that the financial strain from unaffordable housing may negatively affect access to health resources [18].

Three other studies examined the relationship between hypertension and population-level foreclosures. In a cross-sectional study, Chambers et al. found that renters participating in the Hispanic Community Health Study/Study of Latinos living in a high foreclosure risk area (based on census tract-level data) had a higher prevalence of hypertension (PR 1.25, 95% CI 1.08, 1.46) and hypercholesterolemia (PR 1.12, 95% CI 1.01, 1.24) compared to those in medium or low foreclosure risk areas [82]. In a longitudinal study exploring the relationship between hypertension and proximity to foreclosures in the Framingham Offspring Cohort in Massachusetts, Arcaya et al. found that each additional foreclosure located within 100 m of a participant’s home was associated with an increase in systolic blood pressure (SBP) of 1.71 mmHg (95% CI 0.18, 3.24) [81]. In contrast, the longitudinal study by Christine et al. found that an increase in neighborhood foreclosure count of 1.9 foreclosures per quarter mile was associated with a mean decrease in SBP of 0.27 mmHg (95% CI -0.49, -0.04). The authors of this study noted that the variation in their results compared to Arcaya et al. may have been due to differences in measures of foreclosure, different statistical methods, or true variation in different locations. The authors further hypothesized that the stigma associated with foreclosure changed over time, recognizing that the study by Arcaya et al. overlapped with the housing crisis in the mid-2000s [83]. Ultimately, the mixed associations between foreclosures and hypertension in these studies demonstrate that the relationship is complex and depends on several multi-level factors.

### Diabetes

#### Individual-level studies

A relatively larger body of literature (16 quantitative, 3 qualitative studies) exists on housing instability and diabetes outcomes. These studies examined multiple diabetes-related outcomes, including incidence and prevalence, disease control, healthcare utilization, healthcare quality, and self-care behaviors. Two studies analyzed the relationship between housing instability and diabetes incidence or prevalence. A longitudinal study by Schootman et al. of 998 African American adults living in St. Louis, Missouri, found that those reporting fair or poor housing quality (measured by survey questions regarding cleanliness inside building, physical condition of interior, condition of furnishings, condition of exterior of building, and global rating) had higher risk of incident diabetes compared to those reporting good or excellent conditions (cleanliness inside building, OR 1.78, 95% CI 1.03, 3.07; physical condition inside building, OR 2.53, 95% CI 1.47, 4.34; condition of furnishings inside building, OR 2.20, 95% CI 1.29, 3.75; condition of the outside of the building, OR 2.39, 95% CI 1.40, 4.08; overall condition of the dwelling, OR 1.78, 95% CI 1.02, 3.09) [75]. In a cross-sectional study investigating the association between housing instability and diabetes prevalence among white, Asian, and Native Hawaiian/other Pacific Islanders (NHOPIs) in Hawaii, Stupplebeen found that NHOPIs with housing insecurity, measured using a survey question on housing cost burden, had higher adjusted odds of diabetes (adjusted OR 1.85, 95% CI 1.13, 3.01) than those with housing security [76].

Two studies investigated the association between housing instability and diabetes control. In a study of 411 patients with diabetes from four clinics within a practice-based research network in Massachusetts, Berkowitz et al. found in unadjusted analyses that patients with housing instability (measured using survey questions assessing housing status, including homelessness, evictions, frequent moves, or doubling up) were more likely to have poor diabetes control (defined as a composite measure of hemoglobin A1c (HbA1c) > 9% (74.9 mmol/mol), low-density lipoprotein (LDL) cholesterol > 100 mg/dL (2.6 mmol/L), or blood pressure > 140/90 mmHg), but this difference was no longer statistically significant after adjusting for covariates [35, 56]. Similarly, a study of 274,123 adults with type 2 diabetes receiving care at Kaiser Permanente Northern California found that having at least one address change (a potential indicator of housing instability) was associated with higher chance of uncontrolled diabetes (HbA1c > 9% [74.9 mmol/mol], ARR 1.12, 95% CI 1.09, 1.15) and lower chance of controlled diabetes (HbA1c < 8% [63.9 mmol/mol], ARR 0.95, 95% CI 0.94, 0.96) [77].

Three cross-sectional studies assessed the association of housing instability on healthcare utilization in people with diabetes. In the same study by Berkowitz et al. cited above, housing instability was associated with a higher number of outpatient visits after adjusting for covariates (IRR 1.31, 95% CI 1.14, 1.51) [56]. A separate cross-sectional study by Berkowitz et al. examined 1087 nationally-representative, non-homeless, safety-net clinic patients with self-reported diabetes and found that unstable housing (measured using survey responses regarding housing cost burden, frequent moves, and doubling up) was associated with increased diabetes-related emergency department (ED) visits or hospitalizations (adjusted OR 5.17, 95% CI 2.08, 12.87) [35]. The Thomas et al. study mentioned above also found that having at least one address was associated with higher chance of ED visits (ARR 1.25, 95% CI 1.23, 1.27) [77].

Three cross-sectional studies examined diabetes care quality or self-care behaviors. A cross-sectional study of 16,091 employed adults with type 2 diabetes found that housing insecurity (measured using survey responses related to housing cost burden) was associated with decreased likelihood of having a physician visit (adjusted OR 0.58, 95% CI 0.37, 0.92), HbA1c assessment (adjusted OR 0.45, 95% CI 0.26, 0.78), or eye exam (adjusted OR 0.61, 95% CI 0.44, 0.83) [72]. In contrast, Gold et al. examined diabetes guideline-recommended care quality in a cross-sectional study of 73,484 community health center patients with diabetes and found that overall care quality was similar in those with housing insecurity (measured using an unspecified EHR screening tool), except for being less likely to have an up-to-date LDL screening [65]. Vijayaraghavan et al. found that among 711 low-income participants with diabetes, housing instability (ordered into five categories from most to least stable based on survey responses), was significantly associated with decreased diabetes self-efficacy, measured using the validated Self-Efficacy for Diabetes Scale [78].

Four studies assessed housing instability in combination with other adverse social determinants of health to determine the association between a composite measure of unmet basic needs and diabetes prevalence or diabetes-related outcomes. In Heller et al.’s large cross-sectional study mentioned previously, authors found that among adults receiving primary care at an academic medical center in New York, those with three social needs (measured by a survey which included questions on housing quality and instability) was associated with higher prevalence of diabetes (p-trend < 0.001) compared to no needs [54]. Similarly, a cross-sectional study of 5846 adults with type 2 diabetes receiving care from a hospital system based in Bronx, New York found that compared to having no social needs, having three or more needs (based on the same survey used in Heller et al.’s study) was associated with a higher likelihood (adjusted OR 1.59, 95% CI 1.26, 2.00) of uncontrolled diabetes, defined as HbA1c ≥ 9.0% (74.9 mmol/mol). Authors also found that having housing issues (which included problems with both housing quality and housing instability) was associated with higher likelihood of uncontrolled diabetes (*p* < 0.05) [59]. A cross-sectional study of 4043 adult patients with diabetes receiving care at Kaiser Permanente Northwest found that having one or more unmet basic needs (based on a survey which included questions about housing stability and affordability) was associated with an increased odds of having a HbA1c > 8% (63.9 mmol/mol), more outpatient and ED visits, and more delayed refills of diabetes medications compared to having no needs [63]. A study examining the cumulative association of various social risk factors including housing, food, financial, and utility insecurity in 579 adults with diabetes found that those with three or four social risk factors had a greater likelihood of cost-related medication non-adherence, diabetes distress, and anxiety or depression compared to those with no social risks [69].

The association of public housing or government rental assistance with diabetes-related measures was examined in four studies. In the same analysis of the MTO project cited previously, Ludwig et al. found that women from families in the low-poverty voucher group, who were given the opportunity to move out of public housing located in high-poverty census tracts into private-market housing located in low-poverty census tracts, had lower prevalence (− 4.31%, 95% CI − 7.82%, − 0.80%) of HbA1c ≥ 6.5% (47.5 mmol/mol) at a mean follow up of 12.6 years compared to those who received no additional housing assistance [71]. A study using the National Health and Nutrition Examination Survey (NHANES) survey data from 1999–2016 comparing 795 adults receiving either project-based housing (*n* = 450) or housing vouchers (*n* = 345) to 255 adults not yet receiving assistance but remained on the waitlist, found that those receiving project-based housing had lower HbA1c levels compared to the waitlist group, but the differences were not statistically significant. The authors did find, however, that residence in project-based housing was associated with a lower prevalence (− 3.7%, 95% CI − 7.0, 0.0%) of uncontrolled diabetes, defined as HbA1c ≥ 9.0% (74.9 mmol/mol), compared to the waitlist group [61]. A longitudinal cohort study by Lim et al. found that residence in New York City public housing was associated with higher prevalence of stable housing pattern (PR 1.16, 95% CI 1.07, 1.25), based on number of address changes over the 12-year follow-up period, but not with reduced diabetes risk (relative risk [RR] 1.11, 95% CI 0.83, 1.48). Among those experiencing housing instability, living in public housing was associated with a higher risk of diabetes compared to not living in public housing. The authors proposed that one potential mechanism for this finding could be that relocation from one public housing unit to another may cause stress via disruption of social cohesion and support [70]. In the study by Gaston et al., among those in unassisted housing, Black male short sleepers had higher prevalence of diabetes compared to White recommended sleepers, a difference that was not seen among those in government-assisted housing. In women, compared to White recommended sleepers, Black short sleepers had higher prevalence of diabetes in either rental assistance category [64]. As mentioned previously, this finding suggests that rental assistance appears to attenuate racial disparities in diabetes prevalence for men but not women.

Finally, there were three qualitative studies examining both provider and patient perspectives on housing instability and diabetes management. A study on the perspective of providers practicing in Southeastern Appalachian Ohio found that providers cited patients’ housing insecurity, lack of access to providers, lack of access to transportation, food insecurity and financial insecurity as barriers to diabetes care [57]. Two other qualitative studies found that patients with diabetes viewed housing access as an important influence on their diabetes self-management and ability to afford diabetes-related expenses [67] and that transitions to rent-assisted housing may support diabetes self-management [68].

#### Population-level studies

Two quantitative studies examined the relationship between neighborhood-level housing foreclosure and diabetes control. The longitudinal study by Christine et al. used data from the MESA cohort to examine fasting glucose levels and found that an increase in neighborhood foreclosure count of 1.9 foreclosures per quarter mile was associated with an increase in mean fasting glucose of 0.26 mg/dL (0.014 mmol/L; 95% CI 0.04, 0.46) [83]. A longitudinal study by Downing et al. found no statistically significant relationship between changes in foreclosure rate per census-block group and change in annual mean HbA1c level among 105,930 adults with diabetes receiving care at a large integrated healthcare system in Northern California, suggesting that increased foreclosure rates did not worsen glycemic control in this population [85].

### Cardiovascular disease

#### Individual-level studies

Literature examining the association between housing instability and cardiovascular disease (i.e., coronary heart disease, heart failure, and stroke) was limited to four quantitative studies. Three studies analyzed the relationship between individual-level housing instability and cardiovascular disease measures, and one examined the association between cardiovascular disease and government rental assistance use. A study of 2,952,605 Medicare beneficiaries hospitalized for acute myocardial infarction (MI) or congestive heart failure (termed the index admission) found that those with housing instability (defined in this study as two or more unique residential addresses on EHR claims data) had higher odds of hospital readmission within 30 days of discharge from index admission [66]. A large cross-sectional study using data from the Behavioral Risk Factor Surveillance System (BRFSS) to evaluate the independent effects of chronic illness on food and housing insecurity, found that having self-reported cardiovascular disease (i.e., MI, angina, or coronary heart disease) was associated with increased odds of having housing insecurity (OR 1.69, 95% CI 1.07, 2.66), measured using a survey question related to housing cost burden. This study found no association between stroke and housing insecurity. The authors posited that patients experiencing stroke may have more functional limitations that require them to move in with or closer to family members, which in turn increases their level of support and potentially decreases their risk of housing insecurity. In contrast, those with cardiac disease may have higher pharmaceutical costs for medications and decreased likelihood of relocating closer to or moving in with family members compared to patients experiencing stroke, leaving them susceptible to adverse social determinants of health like housing insecurity [60]. The study by Stupplebeen found that among NHOPIs, those with housing insecurity had higher adjusted odds of self-reported MI, angina, coronary heart disease, or stroke [76].

Chambers and Rosenbaum compared cardiovascular disease-related outcomes across three government rental assistance groups (public housing residents, housing vouchers recipients, and people eligible for but not receiving housing assistance) in the cross-sectional Affordable Housing as an Obesity Mediating Environment (AHOME) study of 371 Latino adults. This study found lower odds of cardiovascular disease (defined as having at least one cardiovascular disease [CVD]-related outcome of heart attack, stroke, or hypertension) for those not receiving housing assistance (OR 0.394, 95% CI 0.204, 0.761) and those using housing vouchers (OR 0.527, 95% CI 0.280, 0.992), compared to residents of public housing. They also found that the prevalence of CVD was similar for those using housing vouchers and those not receiving housing assistance. Overall, these findings suggested a potential benefit of housing vouchers use over public housing [58].

#### Population-level studies

Only one study explored the relationship between population-level housing instability and cardiovascular disease. A large cross-sectional study by Segar et al. using data from the American Heart Association’s Get With The Guidelines-Heart Failure registry compared hospital length of stay for heart failure by various social determinants of health and race. This study found that housing instability (measured in this study by ZIP code-level neighborhood/residential characteristics including percentage of housing vacancy, mobile homes, or overcrowding) was associated with longer length of stay for both Black and White adults [91].

## Conclusions

Our review of the literature found generally adverse associations between housing instability and cardiometabolic health conditions of overweight/obesity, hypertension, diabetes, and cardiovascular disease. There is moderate evidence to suggest that housing instability is associated with higher prevalence of overweight/obesity, hypertension, diabetes, and cardiovascular disease, worse hypertension and diabetes control, and higher acute health care utilization among those with diabetes and cardiovascular disease. Most studies included in this narrative review were cross-sectional which do not allow for conclusions to be drawn about the causal direction of these associations. The longitudinal cohort studies produced inconsistent results, and only a few studies leveraged natural experiments to assess the impact of governmental rental assistance use on health outcomes. We found no randomized studies that tested interventions to address housing instability and improve cardiometabolic health.

Through the lens of the conceptual framework proposed in Fig. 1, we can categorize some of the evidence from this narrative review to help understand which pathways may be the best targets for housing policies and interventions aimed at improving cardiometabolic health outcomes. Several studies have shown that housing cost burden is associated with cost-related nonadherence, both to prescription medications and health care visits [5, 18, 69], highlighting the importance of the material budgeting and trade-off pathway in which high housing costs lead to increased financial strain, thereby leaving fewer resources to address health-related needs. While qualitative studies in this review are limited, they have demonstrated that transition to a more stable and affordable housing situation such as subsidized housing frees up financial resources to allow patients to afford health-related expenses [67, 68]. Those with housing instability may also work longer hours or take on additional jobs to offset housing cost burden, leading to decreased time to devote to their health [18]. Additional research is needed to further understand whether obtaining affordable housing through subsidized housing programs can improve adherence and increase patient’s capacity to engage in health promoting behaviors by allowing patients to free up financial resources and time for their health needs.

The second pathway of residential displacement and distribution to poor quality housing and disadvantaged environments may also serve as a crucial target for interventions and policies to improve the health of those with housing instability. The studies in this review which examined the health implications associated with specific types of government rental assistance support the idea that displacement and resultant redistribution of families into disadvantaged neighborhoods can have detrimental health effects. While the overarching purpose of government rental assistance is to alleviate housing cost burden and theoretically improve housing stability, a few studies suggest that transition into subsidized housing, and particularly public housing, is associated with worse cardiometabolic health outcomes [55, 58, 62]. Although transition into public housing may provide housing stability [70], the associated adverse health outcomes may be explained by the fact that public housing units tend to be located in racially segregated areas with high socioeconomic deprivation and limited neighborhood resources [62, 98] which have been tied to poor cardiometabolic health [99, 100]. The MTO demonstration project, a landmark housing mobility study leveraging a natural experimental design, further supported this phenomenon by showing that lifting families out of high-poverty neighborhoods through tenant-based vouchers led to less severe obesity and uncontrolled diabetes [71]. Another study included in this review found that adults receiving tenant-based housing assistance had lower odds of cardiovascular disease compared to those living in public housing [58], suggesting that there may be a benefit of housing voucher programs over unit-based subsidies. Tenant-based assistance allows tenants to rent in the private market which may provide families with more flexibility to choose homes located in better neighborhoods and built environments (i.e., physical characteristics of neighborhoods where people live, work, and recreate), compared to subsidies that are tied specific units and may be located in disadvantaged areas. Housing units located in these areas may suffer from poor housing quality due to lack of community resources and investment, as suggested by another MTO analysis by Nguyen et al. which found that those moving out of public housing in high-poverty areas to private-market housing located in low-poverty areas improved housing quality (i.e. fewer problems with housing units such as broken windows, problems with heating, or pests) compared to those who remained in public housing. While it is well-established that the neighborhood and built environment are associated with cardiometabolic health [47–52], this group of literature suggests that housing quality and neighborhood environment appear to be intimately linked to rental assistance type and clearly play important roles in cardiometabolic health outcomes [49, 52, 71]. Further research, ideally in the form of additional natural experiments, is needed to test differences in cardiometabolic health outcomes by rental assistance type and neighborhood environment, which in turn will help inform policymakers’ prioritization of housing assistance programs.

The third pathway of psychosocial stress and mental health may also help to explain the adverse associations between housing instability and cardiometabolic health. Stress can increase not only in response to one’s own experience of financial strain or a forced move, but also at the population level through observing neighbors’ experiences of residential displacement in areas with high foreclosure and eviction rates, which can contribute to decreased social cohesion and neighborhood disinvestment. In addition to stress, other mental health conditions such as depression, anxiety, and substance use disorder have been linked to housing instability and other frequently coexisting adverse social determinants of health like food insecurity [18, 54, 69, 77]. Efforts to reduce the psychosocial stress and address mental health diagnoses tied to housing instability therefore may improve overall cardiometabolic health, especially since stress, anxiety, and depression have been associated with increased obesity [21, 23, 24], metabolic syndrome [22], diabetes [25, 29, 30], and cardiovascular disease [25–28]. Future research should further define this relationship given its role as an important mediator in the pathway towards improved cardiometabolic health.

While a few themes emerged from our review of this body of literature, a major barrier that precludes further definitive conclusions is the heterogeneity in both the measures used to capture housing instability as an exposure, as well as the cardiometabolic measures examined as outcomes. Housing instability as a construct is variably defined in the literature and can encompass many elements including housing cost burden, overcrowding and doubling up, poor housing quality, frequent moves, forced moves due to evictions or foreclosures, and use of government rental assistance, each of which was measured in various ways throughout this body of literature. Furthermore, each element does not exist in isolation, but rather families often experience multiple housing stressors simultaneously, which likely have more detrimental effects on health than one alone [20]. In addition to the myriad of housing instability measures, the cardiometabolic health outcomes examined in this body of literature also varied widely, spanning domains of disease prevalence and control, healthcare utilization, guideline-recommended care quality, self-management behaviors, and qualitative assessments of provider and patient perspectives on housing and health. Overall, the heterogeneity of current research makes it challenging to identify the most effective housing interventions or policies to improve various aspects of cardiometabolic health.

Although our understanding of the mechanisms driving the adverse associations between housing instability and cardiometabolic health has continued to grow, the complexity of this relationship leaves many gaps in our knowledge and makes it difficult to endorse specific housing policies or programs. Instead, we believe these knowledge gaps highlight potential areas for further research. Given that randomized controlled housing interventions may be difficult, costly, and possibly unethical to design and implement, we recommend that researchers leverage natural experiments to examine the potential impact of new or existing housing policies or programs on cardiometabolic health. Specifically, natural experiments can assess whether programs or policies intended to alleviate housing cost burden, prevent displacement of households into disadvantaged neighborhoods, or address the stress and mental health conditions associated with housing instability have positive effects on cardiometabolic health. This future research will help stakeholders and policymakers focus efforts on existing housing programs, or identify opportunities for new policies in these domains, with the collective goal of improving cardiometabolic health equity.

## Data Availability

Not applicable.

## Abbreviations

AHOME: Affordable Housing as an Obesity Mediating Environment
BMI: Body mass index
BRFSS: Behavioral Risk Factor Surveillance System
CARDIA: Coronary Artery Risk Development In Young Adults
CI: Confidence interval
COVID-19: Coronavirus disease-2019
CVD: Cardiovascular disease
ED: Emergency department
EHR: Electronic health record
GWR: Geographically weighted regression
HbA1c: Hemoglobin A1c
IRR: Incidence rate ratio
LDL: Low-density lipoprotein
MESA: Multi-ethnic Study of Atherosclerosis
MI: Myocardial infarction
MTO: Moving to Opportunity for Fair Housing Demonstration Program
NHANES: National Health and Nutrition Examination
NHOPIs: Native Hawaiian/other Pacific Islanders
OR: Odds ratio
PR: Prevalence ratio
RR: Relative risk
SBP: Systolic blood pressure
VA: Veterans Health Administration
ZIP: Zone improvement plan
